# Friction and Wear between Polymer and Metal in the Mixing Process

**DOI:** 10.3390/ma12244029

**Published:** 2019-12-04

**Authors:** Yiren Pan, Lin Zhu, Huaqiao Liu, Meng Zhang, Wenwen Han, Chuansheng Wang, Huiguang Bian

**Affiliations:** 1 College of Electromechanical Engineering, Qingdao University of Science and Technology, Qingdao 266061, China; pyr90hot@163.com (Y.P.); qustzhulin@163.com (L.Z.); cm0004@tta-solution.com (H.L.); zhangmeng96710@163.com (M.Z.); hbhanwenwen@163.com (W.H.); 2 National Engineering Laboratory for Advanced Tire Equipment and Key Materials, Qingdao University of Science and Technology, Qingdao 266061, China

**Keywords:** mixing chamber, carbon black, mixing process, metal, friction and wear

## Abstract

In order to obtain a longer mixing chamber life, a layer of hard alloy coating is generally welded on the surface. However, when the mixing chamber is used for a long time, the surface will be worn due to friction with small fillers and rubber. As a result, there will be a large gap between the mixing chamber and the rotor, which will further affect the quality of the mixed rubber. In this paper, the dispersion process of the reinforcing system is simulated at first, and the mixed rubber samples are obtained from different dispersion stages in preparation for experiments with the chamber material. On this basis, the friction experiment is carried out with the same material as the mixing chamber on the friction experiment machine employed in the improved test part. The experiment shows that the friction and wear between the mixture and metal produced in each mixing stage are different. The wear in the stage with high friction is not necessarily large. The wear will be intensified in the middle and later mixing periods, while the friction will tend to be stable. In this paper, besides the exploration on the friction of fillers and rubber on the mixing chamber in different mixing stages, the most important thing is to change the mixing process of rubber formula, so as to extend the service life of the mixing chamber without changing the comprehensive physical properties of the mixing rubber.

## 1. Introduction

The physical and mechanical properties of rubber are closely related to the addition of carbon black. As an important reinforcing agent and filler, carbon black can obviously improve the strength of rubber and reduce the cost of rubber material [1,2,3]. The dispersion of carbon black in rubber will directly affect the subsequent processing performance and comprehensive physical properties of the rubber product. In order to improve the distribution and dispersion of carbon in rubber formulae, the mixer becomes the most critical equipment [4].

**Figure 1:**
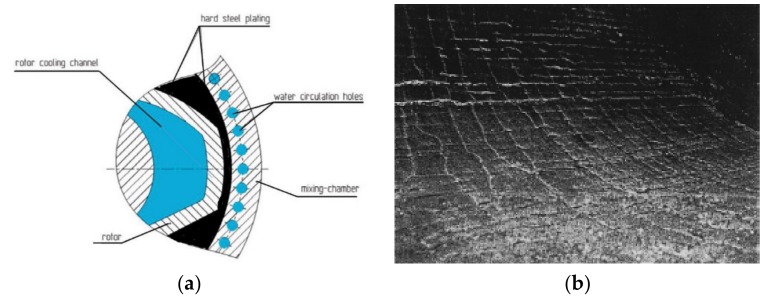
Mixing process and chamber wear diagram: (**a**) Chamber structure and (**b**) photo of the chamber wear.

The chamber is an important key part of the mixer. As we know, friction is a double-edged sword. On the one hand, it plays a vital role in mixing and, on the other hand, it can cause metal wear. In the mixing process, when the compound passes between the rotor and the wall of the chamber, the chamber wall will be subjected to intense friction and extrusion from the compound, and the stress is complex. Therefore, the wear of the compound on the mixer wall is also very serious. When the inner wall of the mixing chamber is worn, the gap between the rotor and the inner wall of the mixing chamber has a tendency to expand, resulting in an uneven gap and different sizes. In this case, the rubber will face uneven shear force, which cannot guarantee the dispersion and distribution of carbon in the rubber [5,6,7,8,9]. The abrasion of the chamber wall is shown in Figure 1b.

**Figure 2:**
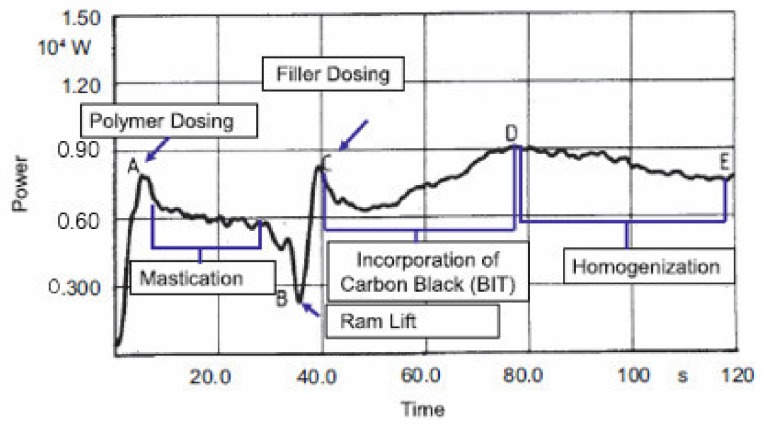
Power curve of mixing.

In past experiments it was found that the power curves of whole mixing process was different when they were added to the mixer, and the mixing curve is shown in Figure 2. Dolezal et al. used the so-called “power graph” composed of the power curve of the mixing process and the change curve of the rubber properties as a method to study the mixing process. Hetzel et al. used the power curve and the performance of the compound to select the mixing process conditions. In other words, the friction and wear of the mixer are different in each mixing stage. Therefore, this paper mainly starts from the whole mixing process to explore the change of friction and wear between carbon black rubber composite and metal at every stage.

Therefore, in this paper, EDEM discrete element simulation analysis software (version 2.7, DEM Solutions, Edinburgh, UK) is firstly used to simulate the mixing process [10]. Then, during a common mixing process, samples are selected from different stages. At the same time, we try to maintain the consistency of the test environment, including temperature and humidity. In this paper, simulation and experiments are combined to study the effects of different stages on the friction and wear of the mixing chamber. In the study process, we also focus on finding the stages with the most severe friction and wear on the mixing chamber and, from the point of view of technology, to find a way to reduce wear.

## 2. Materials and Methods 

### 2.1. EDEM Simulation Parameters

**Figure 3:**
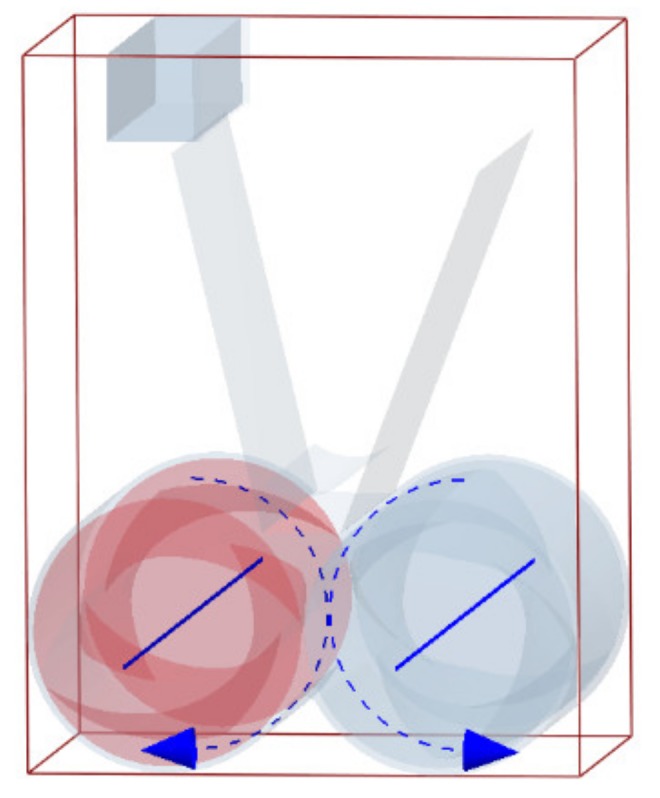
Simulation model.

This model adopts the rotor of the isotropic synchronous shear mixer. The model is shown in Figure 3.

#### 2.1.1. Simulation Parameters

In this paper, there are three kinds of materials, namely, two kinds of granular materials and one geometric model material. In the calculation process, there is contact between particles, as well as between particles and geometry. The calculation of the contact force is based on the contact model. Due to the viscoelasticity of the rubber particles themselves, the particle model adopted is the soft ball model, while the Hertz–Mindlin model with the JKR (Johnson-Kendall-Roberts) cohesion model was used between rubber and rubber particles [11,12,13,14,15,16]. In the mixing process, carbon black itself does not have viscosity, but under the action of the rotor, the rubber was constantly deformed and carbon was covered by rubber, so that the carbon black particles can move with the rubber and finally disperse evenly. Therefore, the Hertz–Mindlin model with JKR cohesion was used between the rubber particles and carbon particles, while the Hertz–Mindlin (no slip) model was used between carbon and carbon particles. We set the red particles as rubber and the blue particles as carbon, the rubber particle radius is set at 0.5 mm, and the carbon particle radius is set at 0.4 mm. Moreover, viscosity parameters are set between red particles, but not between blue particles. Additionally, the viscosity parameter between red particles is 4J/m^2^ and the viscosity parameter between red particles and blue particles is 2 J/m^2^.

#### 2.1.2. Simulation Mathematical Model

In the case of using discrete element method for simulation, the vibration motion of rubber particles and carbon particles is decomposed into normal and tangential directions. The equation of normal vibration motion is:

(1) $$m^{*}d^{2}u_{n}/dt^{2}+c_{n}du_{n}/dt+k_{n}u_{n}=F_{n}$$

Tangential motion is characterized by tangential sliding and particle rolling:

(2) $$m^{*}d^{2}u_{s}/dt^{2}+c_{t}du_{t}/dt+k_{t}u_{t}=F_{t}$$

(3) $$I^{*}d^{2}\theta /dt^{2}+(c_{t}du_{t}/dt+k_{t}u_{t})s=M$$

where m* is the equivalent mass of particles; I* is the equivalent transmission inertia of particles; $u_{n},\;u_{t}$ are the relative displacements in the normal and tangential directions of the particles;$\;c_{n},\;c_{t}$ are the damping coefficients in the normal and tangential directions of the contact model;$\;k_{n},\;k_{t}\;$ are the elastic coefficients in the normal and tangential directions of the contact model; $\;F_{n},\;F_{t}$ are the forces in the normal and tangential directions of the particles; s is the turning radius; θ is the rotation angle of particle; and M is the external torque on the particles.

The contact model used in this article is based on the soft ball model, where the Hertz–Mindlin with JKR cohesion model was used between particle and particle, and the Hertz–Mindlin (No Slip) model was used between particles and geometry.

The calculation of the JKR normal force, *F_JKR_* is based on the amount of overlap of *α* interaction parameters and surface energy *ϑ*:

(4) FJKR=−4πϑE*a32+4E*3R*a3

(5) α=σ2R*−4πϑσE*

The Hertz–Mindlin (No Slip) model is the default contact model in EDEM, which has high computational efficiency. In this model, both normal and tangential forces have damping forces, and tangential friction follows Coulomb’s Friction Law.

In this model, the normal force Fn is calculated based on the overlap *α* amount:

(6) Fn=43E*R*αn32

where *α_n_* is the normal amount of overlap (if the contact model is a soft ball model, the two particles will overlap when they contact).

Damping force in the normal direction $F_{n}^{d}$:

(7) $$F_{n}^{d}=-2\sqrt{\frac{5}{6}}\beta \sqrt{S_{n}m^{*}}v_{n}^{\vec{rel}}$$

where $m^{*}$ is the equivalent quality and $\;v_{n}^{\vec{rel}}$ is the normal component of relative velocity of particles.

### 2.2. Materials

NR (TSR20, Thailand 20# Standard Rubber), (Nakhon Si Thammarat, Thailand); BR (BR9000), Products of PetroChina Dushanzi Petrochemical Company (Fushun, China); Carbon Black: N375, Cabot, Boston, MA, USA, the physical properties are shown in Table 1.

### 2.3. Preparation and Testing of Rubber Compounds

#### 2.3.1. Experimental Formulation

The formula employed in this study is shown in Table 2. Mixing was carried out using the Hackmie machine developed by the Qingdao University of Science and Technology (Qingdao, China). The fill factor and rotor speed were kept constant at 0.75 and 80 rpm, respectively. In this experiment, rubber and compound materials were mixed depending on Table 2. Due to the carbon formula, the temperature of the rubber discharging was 140–150 °C. In the mixing process, samples were collected in different stages, including the very beginning process, adding of carbon black, and the mixing of carbon black and rubber evenly.

#### 2.3.2. Experimental Process

Compounds samples were prepared according to the time in Table 3.

**Figure 4:**
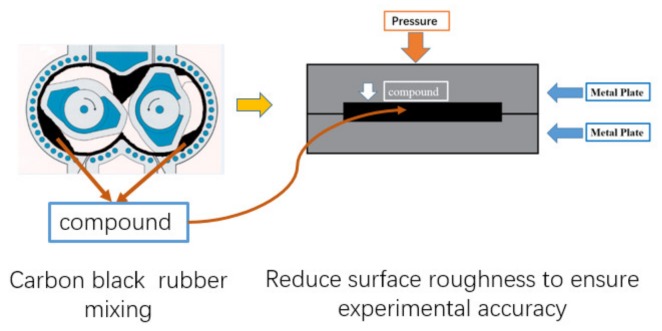
Sample preparation process.

Due to the requirements on the shape of the rubber samples in the experiment, and to ensure the accuracy of the experiment, the test compound samples made will be pressurized through a steel plate. In the test, the friction coefficient is related to the surface roughness, and the mixing degree of carbon black and rubber is the important factor affecting the surface roughness. In particular, the friction coefficient tended to be stable due to carbon black gradually dispersing evenly in the rubber during the later mixing period. However, the surface roughness of the rubber test samples is still different. This is the uncertainty factor which affects the measurement of the friction coefficient in the experiment. Therefore, before the friction test, we put the compound samples into a grinding tool to make the surface roughness as smooth as possible to reduce the test error. The production process of the test piece is shown in Figure 4.

**Figure 5:**
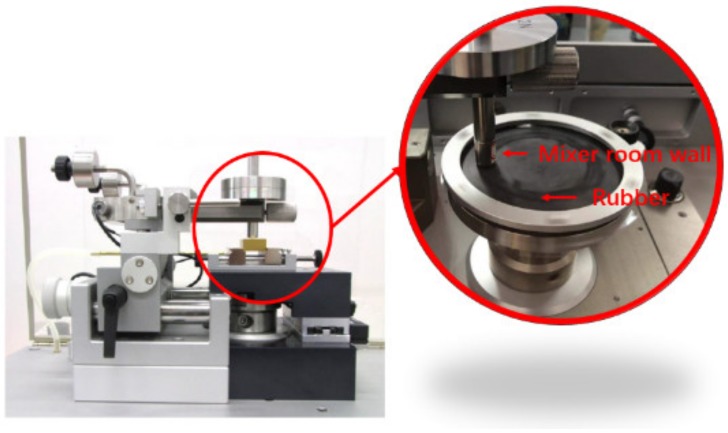
Equipment improvement image.

Friction and wear experiments between carbon black rubber composites and metal were carried out by tribometer (Antor Paar, Buchs, Switzerland), and a CSM test for short (shown in Figure 5). After the calibration, the column plate model was chosen. Based on the calculation, we set the experimental pressure as 3 N, and the experiment time as half an hour. In order to accurately test the friction between the rubber and the wall of the mixing chamber during the mixing process, the mechanical test part of the equipment was improved to make the test contact surface the same as the material of the mixing chamber. The friction test joints can be replaced many times to repeat the test to ensure the accuracy of the experiment.

Three-dimensional morphology test: An Olympus 4500 (Three-dimensional topography measuring instrument, Japan) was used to observe and measure the friction and wear of the metal surface.

## 3. Results and Discussion

### 3.1. EDEM Simulation and Experimental Results of the Mixing Process

**Figure 6:**
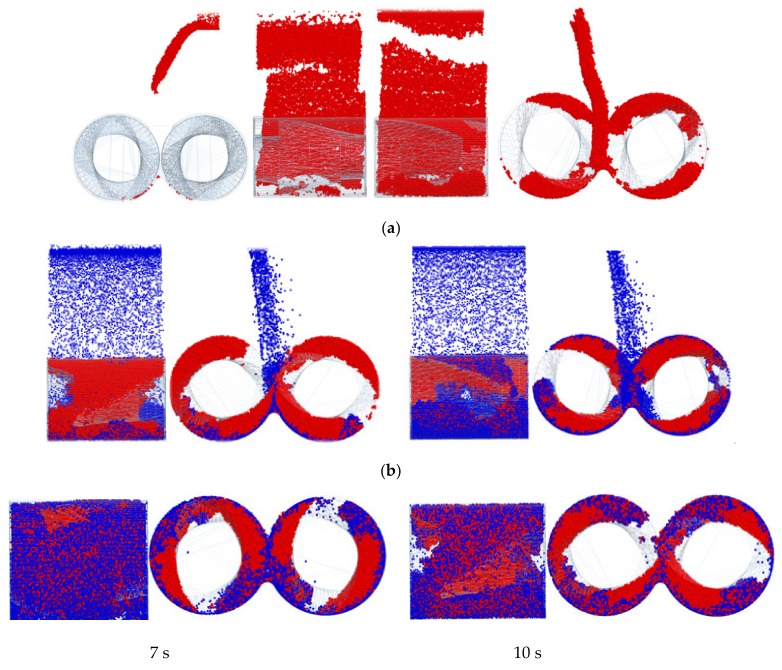

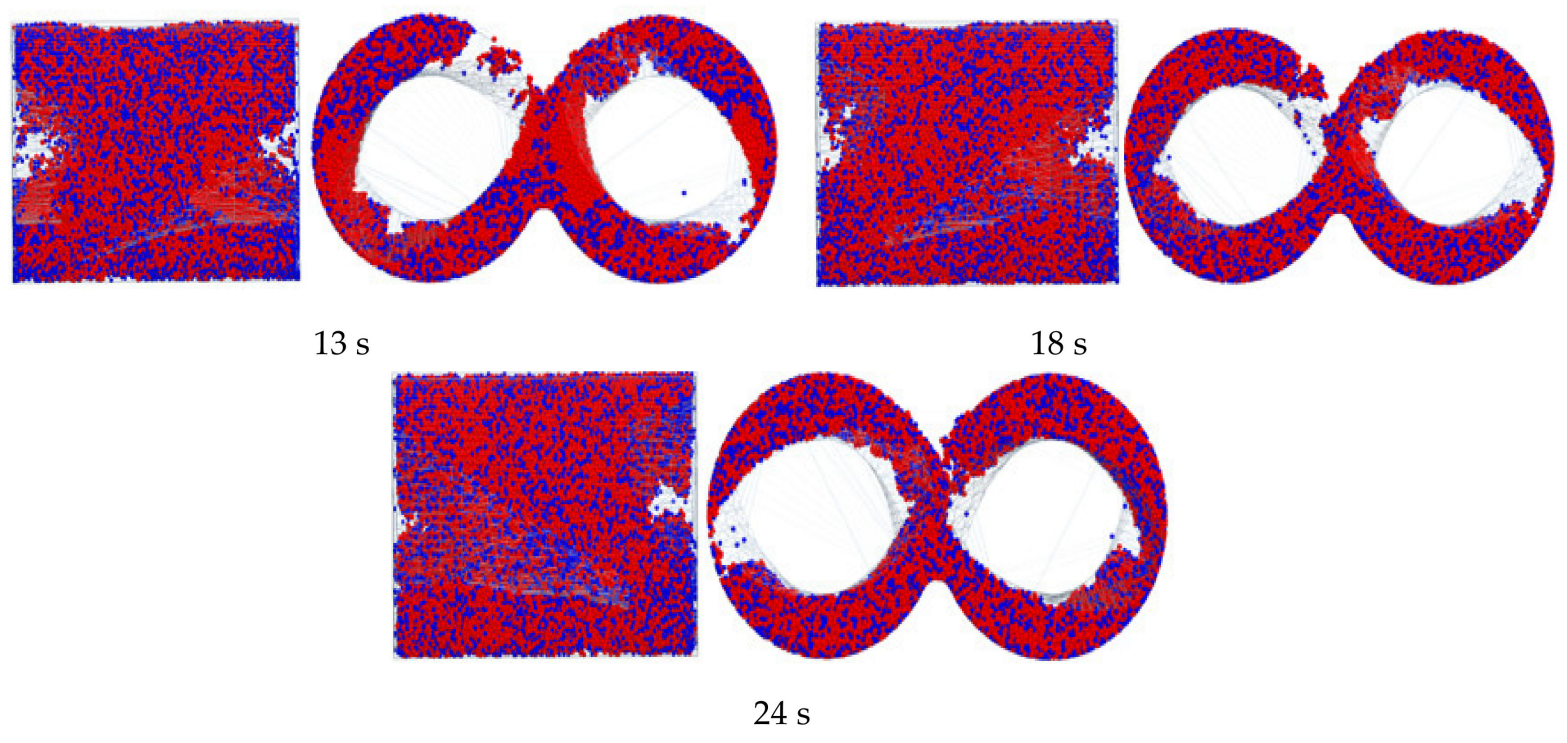
Dispersion simulation of carbon black at each stage of the mixing process. (**a**) Rubber plastic process; and (**b**) carbon added.

Figure 6 shows the dispersion simulation of carbon black at each stage of the mixing process. In the mixing process, the dispersion and distribution of carbon in each stage of rubber mixing are different. According to the simulation results, it is found that when carbon was added, most of the initial accumulation would occur between the rotor edges and the chamber walls, especially the chamber edges and the chamber bottom. As the mixing process continues, the large aggregate of carbon is transformed into small aggregate. There is an interesting phenomenon from the simulation that there are many carbon particles or small aggregate at the edge of the chamber. When the mixing process has been going on for some time, the aggregate becomes smaller and is broken up, and then it starts to disperse internally from the chamber edges. At the end of the mixing process, carbon particles or small carbon aggregates are dispersed evenly in the rubber. According to the simulation results of the dispersion of carbon in rubber during the whole mixing process, the mixing stage with the most serious friction and wear appears when the carbon is added to the mixer with little mixing time. When the carbon is evenly and uniformly dispersed in the rubber, the friction and wear will present a balanced state.

### 3.2. Test Results

**Figure 7:**
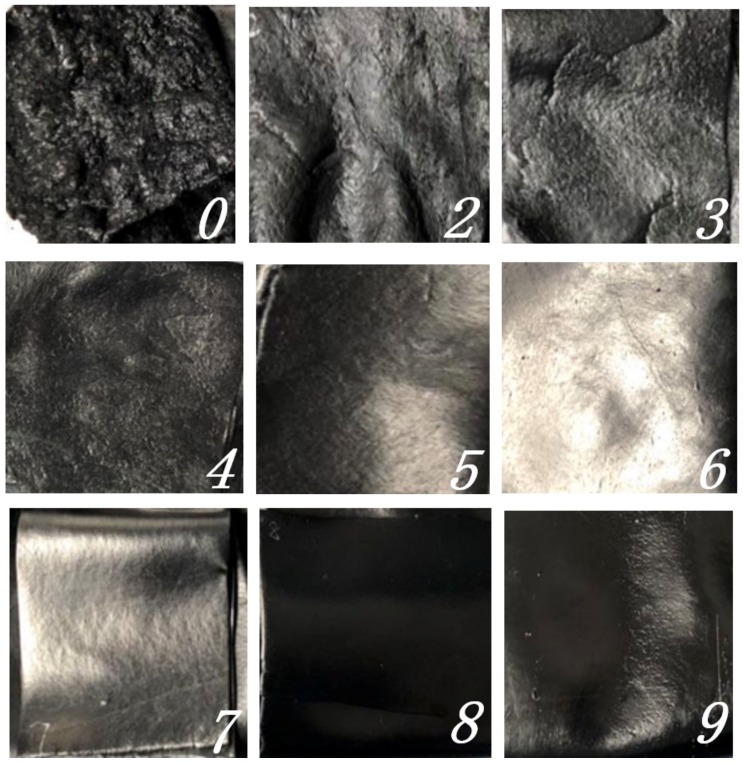
Samples from nine stages.

In order to verify whether the conjecture obtained through simulation is correct, samples were taken at different stages of mixing and put on the CSM test machine for friction and wear experiments. Sample photos are shown in Figure 7.

**Figure 8:**
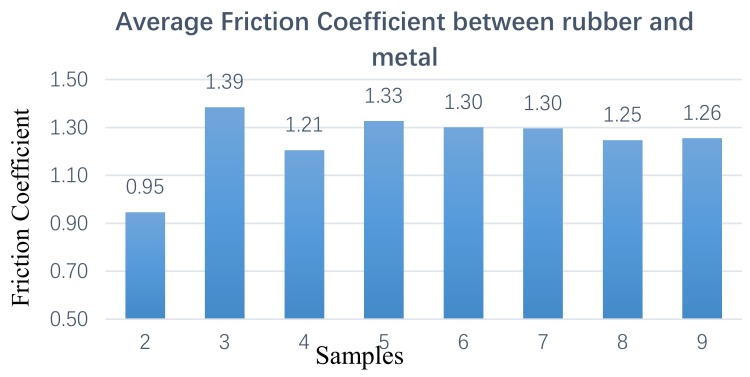
Average friction coefficient.

Nine groups of samples were prepared for the CSM friction test, and the friction curve and friction coefficient were obtained as shown in Figure 8 (the aggregate of sample 1 is too large, so it is prone to fracture in the friction experiment; therefore, the data of sample 1 is temporarily excluded).

Figure 8 shows the friction coefficient between the rubber and the metal obtained by the CSM test. From the test data in Figure 8, it can be seen that, at each stage, the friction coefficient between the sample and metal is different. The change trend of the friction coefficient is not a single increase, which would have a peak and a flat area.

**Figure 9:**
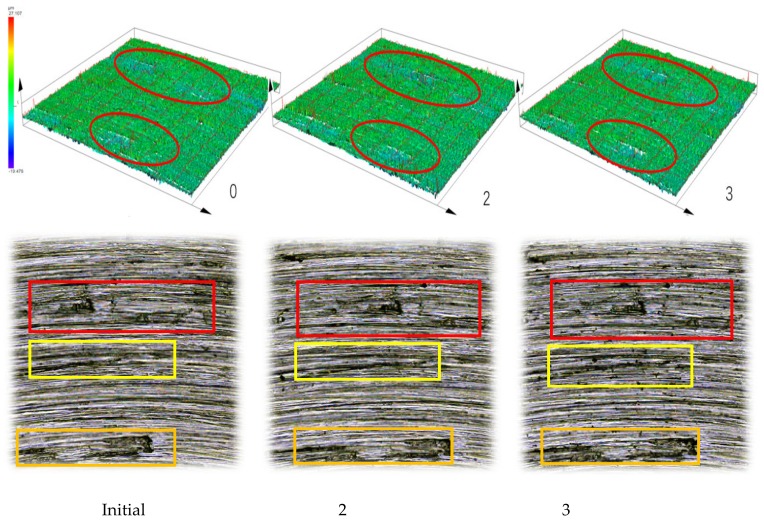

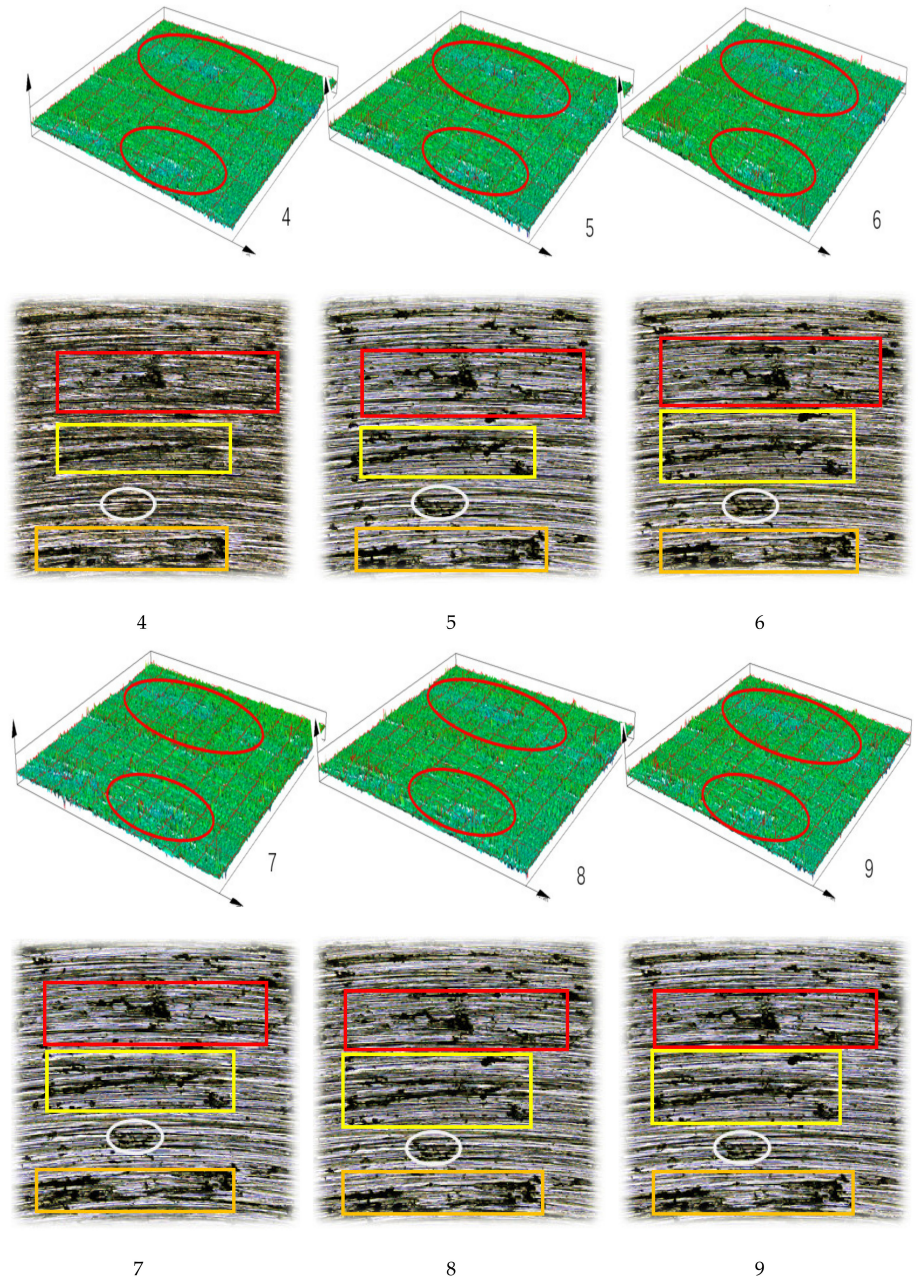
Surface topography observation of metal in different stages of friction.

After the rotary friction experiment, the wear surface of the metal samples was observed with an Olympus confocal microscope, as shown in Figure 9.

Figure 9 shows that the same location was set for observation, and the three-dimensional image combined with the two-dimensional images were measured with the confocal microscope, so as to measure the volume, V, surface roughness, Sa, surface mean root deviation, Sq, and surface roughness for further comparison. The data is shown in Table 4.

Through the comprehensive analysis of the friction curve, friction coefficient, three-dimensional shape, as well as two-dimensional pictures during the friction process, it is found that in the whole mixing process, when rubber is added to the mixing chamber for plastic refining, the friction between the rubber and the mixing chamber belongs to dry friction. However, because of the properties of the raw rubber and the temperature produced by the mechanical force, the rubber surface will soften and change from dry friction to boundary lubricating friction.

It can be seen from Table 4 that when carbon black is added to the mixer, the friction coefficient is relatively smaller than at other stages. From the 2D and 3D surface figures obtained from the confocal microscope, there is no obvious amount of wear at this stage. Generally speaking, the friction coefficient and wear should be increased because of the increase in the volume filling amount when carbon black is added to the mixer. However, the opposite conclusion was obtained through the test in this paper. This phenomenon can be explained in that the addition of carbon black plays a role in lubrication to reduce friction and wear between rubber, the carbon black compound, and metal. The reason for this phenomenon is that existing carbon black is mainly prepared by the furnace method, in which some industrial oil is retained on the surface, which means that there is a layer of oil film on the surface. It can be verified that there is an oil film on the carbon black particles’ surface by the light transmittance experiment of toluene, as shown in Table 5.

Since the raw oil on the surface of carbon black is mainly polycyclic aromatic hydrocarbon, it can only be extracted by solvent, such as toluene or acetone. The test data of toluene transmittance in Table 4 is 87%, which can indirectly indicate that part of polycyclic aromatic hydrocarbons on the surface of carbon black are extracted, and verify the existence of oil film on the surface of carbon black particles. This is why the friction coefficient of sample no. 2 is lower than other stage. The simulated motion track shows that when carbon black particles are added to the mixing chamber, with the rotation of the rotor, carbon black particles will disperse to all corners of the mixing chamber. When it passes between the rotor and the chamber wall, the oil film is slowly split under the action of shear tension. Under the protection of this oil film, the friction coefficient decreases, just as shown in Table 4.

As the mixing continues, the oil film of carbon black particles surface is gradually absorbed. The rubber is wetted and makes full contact with the carbon black surface through flow or deformation, and penetrates into the interior of carbon black structure. This process is called the infiltration stage. Through EDEM simulation results, rubber particles and carbon black particles begin to mix with each other at this stage. At this time, carbon black is still in the large aggregate form and the friction coefficient increases. According to the metal surface appearance 2D figures which were observed by the confocal microscope, it is clear that metal wear begins to appear. As the infiltration process continues, the internal voids of the mixed system are gradually reduced, while more and more raw rubber penetrated into the carbon black and, finally, a high concentration of raw rubber-carbon black agglomeration was formed. At this time, the friction between the metal and the rubber plays a role in promoting the mixing, but the wear on the metal surface is intensified. This process can be seen in panel 4 of Figure 9.

With the development of mixing, and under the action of tensile shear between the rotor and rotor, as well as between rotor and chamber wall, carbon black rubber aggregates are broken and separated, then dispersed into the whole rubber system. This process is constantly changing and repeated, and the large aggregate gradually becomes a small aggregate with uniform dispersion, as shown in Figure 8. In this stage, the mixing temperature begins to rise, and the carbon black gradually disperses evenly in the rubber. Combined with the 2D images of the metal surface topography measured by the confocal microscope, and the analysis of the volume and surface roughness obtained in panels 5–7 of Figure 9, it is found that the surface roughness tends to be flat, but the volume and surface area are relatively reduced. This indicates that the wear between the compound and the metal is intensified when the large carbon black aggregate is gradually broken and evenly mixed into the rubber. At the same time, the temperature rise leads to the compound’s viscosity increase, and the adhesion friction intensifies the wear between the rubber and metal

**Figure 10:**
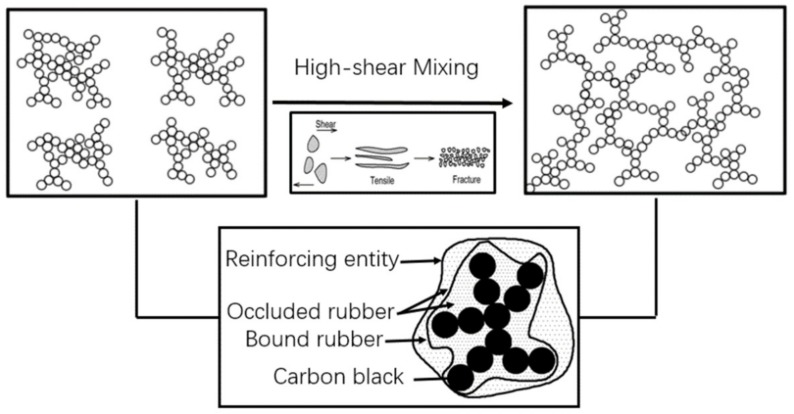
Dispersion and distribution of carbon black aggregates.

At the later stage of mixing, carbon black is uniformly dispersed and distributed in the rubber. At this time, carbon black particles have been fully wrapped by the rubber and form rubber–carbon black inclusions, which was shown in Figure 10. At this stage, the oil film of the carbon black has been fully absorbed. According to the actual data provided by the factory, the mixing temperature of the carbon black formula should be maintained at 140–145 °C for about one minute to ensure better dispersion of carbon black and discharge temperature. Therefore, under this temperature, friction and wear between the rubber and metal are mainly caused by adhesion and lag friction. According to the EDEM simulation analysis, carbon black is dispersed evenly in the rubber and its volume remains constant at the later stage of mixing, so the friction coefficient decreases relatively, and the wear of the metal surface remains stable compared with the previous stages.

## 4. Conclusios

This paper mainly studies the friction and wear phenomenon between a carbon black compound and metal during mixing. Combined with discrete element simulation analysis and experimental results to analyze the change of the friction and wear in the mixing process, the experimental results show that the friction and wear between the rubber and the metal do not increase singly from the time when the raw rubber is added to the mixer for plastic refining to when the carbon black is dispersed and mixed evenly in the rubber. Combined with the properties of rubber and carbon black, during the whole mixing process, the friction and wear between the carbon black rubber composites and the metal will change with the carbon black dispersion in the rubber. At the same time adhesion and hysteresis friction of the compounds caused by the temperature rise will also aggravate the metal wear, while the friction coefficient does not increase at this stage. At the end stage of mixing, the friction coefficient and wear tend to be stable. Through experiments, there are three stages of serious friction and wear: 1) carbon black and rubber begin to immerse each other; 2) the stage of carbon black rubber aggregates are broken and separated; and 3) the stage of adhesion and lag friction caused by the temperature rise.

As is known, a certain amount of environmentally-friendly oil will be added to the rubber tire formula, its main purposes are: 1) to speed up the filler mixing into the rubber; and 2) to reduce the hardness of the rubber products to obtain its special properties. Combined with the experimental results, we think that the environmentally-friendly oil can be added in two serious wear stages of the chamber to replace the original oil addition process. In this way, it not only accelerates the carbon black mixing into the rubber, but also prolongs the service life of the mixing chamber.

## Figures and Tables

**Table 1:** Physical properties.

Test List	Indicators Project
Iodine Adsorption Number (g/kg)	90 ± 6
DBP (10^−5^ m^3^/kg)	114 ± 6
CATB (10^3^ m^2^/kg)	90–102
Ash %≤	0.7

**Table 2:** Formulation.

List	Phr
TSR20	50
BR9000	50
N375	58
Sum	158

**Table 3:** Mixing process and sampling time.

1.6 L hake mixer, 80 rpm, 75% FF		
Time	T (°C)	Ingredients
Master batch		
0:00	70	Polymers
0:40	-	Half carbon
1:20	-	Another half carbon, take sample
1:40	110	Take sample
2:10	120	Take sample
2:40	130	Take sample
3:10	140	Take sample
3:40	140	Take sample
4:00	140	Take sample

**Table 4:** Friction surface test data.

Test List	Volume (mm^3^)	Superficial Area (mm^2^)	Surface Roughness (μm)
0	0.75	37.95	1.738
2	0.743	38.09	1.697
3	0.672	35.73	1.684
4	0.525	26.52	1.672
5	0.477	26.44	1.721
6	0.490	23.08	1.703
7	0.456	22.69	1.694
8	0.529	28.60	1.70
9	0.536	28.64	1.704

**Table 5:** Light transmittance experiment of toluene of N375.

Sample	Determination of Light Transmittance of Toluene %
N375	87%

## References

[1] Yang Q.Z. (2001). Modern Rubber Technology.

[2] Wang C.S., Li Z., Kan L.L. (2010). The effect of carbon black dispersion on physical and mechanical properties of rubber compound. China Rubber/Plast. Technol. Equip..

[3] Zhang H.X., Liu Y.D. (1985). Effects of carbon combined with rubber on properties of natural rubber. Appl. Chem..

[4] Wang M.J. (1998). Effect of Polymer-Filler and Filler-Filler Interactions. Rubber Chem. Technol..

[5] Kim M.H., White J.L. (1992). Simulation of Flow in a Farrel Continuous Mixer. Int. Polym. Process..

[6] Inoue T., Soen T., Hashimoto T., Kawai H. (1970). Studies on Domain Formation of the A-B-Type Block Copolymer from Its Solutions. Ternary Polymer Blend of the Styrene-Isoprene Block Colopymer with Polystyrene and Polyisoprene. Macromolecules.

[7] White J.L. (1995). Rubber Processing Technology Materials Principles.

[8] Nicholasp C. (1987). Polymer Mixing and Extrusion Technology.

[9] Stein R.S., Khambatta F.B., Warner F.P., Russell T., Escala A., Balizer E. (1978). X-ray and optical studies of the morphology of polymer blends. Polym. Sci. Polym. Symp..

[10] Payne A.R. (1962). The Dynamic Properties of Carbon Black Loaded Natural Rubber Vulcanizates. Part I. J. Appl. Polym. Sci..

[11] Hongxiang T., Rui S., Yan D., Xiaoyu S. (2019). Measurement of Restitution and Friction Coefficients for Granular Particles and Discrete Element Simulation for the Tests of Glass Beads. Materials.

[12] Zhang H.Q. (2009). Analysis and improvement of main assembly and wear clearance in mixing chamber of F 370 mixer. Guangxi J. Light Ind..

[13] Nakajima N. (1980). Elongational Flow in Mixing Elastomer with Carbon Black. Rubber Chem. Tech..

[14] Min K., Suh K.G. (1991). Experiments and Modeling of Flow of Elastomers in an Internal Mixerwith Intermeshing Rotors. Polym. Eng. Sci..

[15] Chen G., Schott D.L., Lodewijks G. (2017). Sensitivity analysis of DEM prediction for sliding wear by single iron ore particle. Eng. Comput..

[16] Dhakal P., Das S.R., Poudyal H., Chandy A.J. (2017). Numerical simulations of partially-filled rubber mixing in a 2-wing rotor-equipped chamber. J. Appl. Polym. Sci..

